# Comparative proteomic analysis of hypertrophic chondrocytes in osteoarthritis

**DOI:** 10.1186/s12014-015-9085-6

**Published:** 2015-04-25

**Authors:** Konstantinos C Tsolis, Ekaterini S Bei, Ioanna Papathanasiou, Fotini Kostopoulou, Vassiliki Gkretsi, Kalliopi Kalantzaki, Konstantinos Malizos, Michalis Zervakis, Aspasia Tsezou, Anastassios Economou

**Affiliations:** Institute of Molecular Biology and Biotechnology – FoRTH, Iraklio, Greece; Department of Microbiology and Immunology, Rega Institute for Medical Research, KULeuven, Leuven, Belgium; School of Electronic and Computer Engineering, Technical Univ. of Crete, Chania, Greece; Department of Biology, University of Thessaly, Faculty of Medicine, Larissa, Greece; Institute for Research & Technology-Thessaly/Centre for Research & Technology-Hellas (CE.R.T.H), Larissa, Greece; Department of Orthopedics, University of Thessaly, Faculty of Medicine, Larissa, Greece

**Keywords:** Osteoarthritis, Cartilage, Chondrocytes, Proteomics, Mass spectrometry, Pathway analysis, PLS3, GSTP1

## Abstract

**Background:**

Osteoarthritis (OA) is a multi-factorial disease leading progressively to loss of articular cartilage and subsequently to loss of joint function. While hypertrophy of chondrocytes is a physiological process implicated in the longitudinal growth of long bones, hypertrophy-like alterations in chondrocytes play a major role in OA. We performed a quantitative proteomic analysis in osteoarthritic and normal chondrocytes followed by functional analyses to investigate proteome changes and molecular pathways involved in OA pathogenesis.

**Methods:**

Chondrocytes were isolated from articular cartilage of ten patients with primary OA undergoing knee replacement surgery and six normal donors undergoing fracture repair surgery without history of joint disease and no OA clinical manifestations. We analyzed the proteome of chondrocytes using high resolution mass spectrometry and quantified it by label-free quantification and western blot analysis. We also used WebGestalt, a web-based enrichment tool for the functional annotation and pathway analysis of the differentially synthesized proteins, using the Wikipathways database. ClueGO, a Cytoscape plug-in, is also used to compare groups of proteins and to visualize the functionally organized Gene Ontology (GO) terms and pathways in the form of dynamical network structures.

**Results:**

The proteomic analysis led to the identification of a total of ~2400 proteins. 269 of them showed differential synthesis levels between the two groups. Using functional annotation, we found that proteins belonging to pathways associated with regulation of the actin cytoskeleton, EGF/EGFR, TGF-β, MAPK signaling, integrin-mediated cell adhesion, and lipid metabolism were significantly enriched in the OA samples (p ≤10^−5^). We also observed that the proteins GSTP1, PLS3, MYOF, HSD17B12, PRDX2, APCS, PLA2G2A SERPINH1/HSP47 and MVP, show distinct synthesis levels, characteristic for OA or control chondrocytes.

**Conclusion:**

In this study we compared the quantitative changes in proteins synthesized in osteoarthritic compared to normal chondrocytes. We identified several pathways and proteins to be associated with OA chondrocytes. This study provides evidence for further testing on the molecular mechanism of the disease and also propose proteins as candidate markers of OA chondrocyte phenotype.

**Electronic supplementary material:**

The online version of this article (doi:10.1186/s12014-015-9085-6) contains supplementary material, which is available to authorized users.

## Background

Osteoarthritis (OA) is the most common form of arthritis and a major cause of disability worldwide. OA affects the whole joint, leading to cartilage degradation, synovial inflammation and subchondral bone remodelling [1,2]. Disease progression is slow. Structural alterations start before middle age but can be diagnosed when they become symptomatic where at that time there is a severe damage in the joint. The aetiology of the disease is not completely defined [1-5]. Several patho-physiological processes are involved in the disease phenotype, however the triggering event for disease onset is unknown, as a result OA is considered a multi-factorial disease [1,3,6-8]. Several risk factors have been associated with OA, including age, obesity, mechanical load, genetic predisposition, sex, and prior joint injury [7,9].

Currently, there is not efficient treatment for OA. Recommended pharmacological interventions aim for the relief of pain and joint inflammation by administration of mild analgesics first and non-steroid anti-inflammatory drugs (NSAIDs), later [10,11]. Intra-articular injection of hyaluronic acid (HA) or corticosteroids was also shown to improve pain, whereas the HA administration showed more prolonged effect [10,11]. Another approach that is currently under research for the management of OA, is the use of disease-modifying osteoarthritis drugs (DMOADs) (e.g. bisphosphonates, doxycycline, strontium ranelate) [12,13]. This category of drugs slows down the cartilage degradation, stalling or reducing the disease progression. Other approaches aiming for the regeneration of cartilage, make use of growth factors or cytokines, either by direct injection to the infected areas or using platelet-rich plasma or gene therapy approaches [10,13-15]. All these methods were tested in animal models and clinical trials, however further studies are needed to verify their efficacy and the prerequisites in which these approaches could be beneficial. Collectively, the latest approaches for the management of OA highlight the importance of unravelling the molecular mechanism of OA progression, in order to design more targeted and effective therapeutic strategies.

Chondrocytes comprise the cell population responsible for cartilage homeostasis. During OA progression, chondrocytes acquire a hypertrophic phenotype, showing increased synthesis of several markers including COL10A1, MMP-13 and Runx2 [16]. Even though there is a wealth of information about chondrocyte differentiation mechanisms, it is not completely understood how chondrocytes are affected during the progression of osteoarthritis [16-18]. Different -omics approaches have been used for the identification of the alterations that occur in articular chondrocytes during OA progression. Previous proteomics studies highlighted mitochondrial dysfunction and redox imbalance in osteoarthritic chondrocytes [19,20]. Another study, combining genomics and lipidomics analysis revealed that OA articular chondrocytes generate multiple eicosanoid products which show a complex role in cartilage homeostasis and pathophysiology of the disease [21].

The aim of this study was to improve our understanding of the molecular basis of OA by performing a comparative proteomic analysis of articular chondrocytes derived from patients with OA and from disease-free individuals, using high-resolution mass spectrometry and label-free quantification. 269 proteins showed significantly differential synthesis between patients and controls and these were functionally annotated and linked through network analysis, for the identification of substantially enriched pathways, related with the disease. Pathway analysis showed enrichment in those related to the cytoskeleton, adhesion and lipid metabolism. Also, we propose that eight of the identified proteins (GSTP1, PLS3, MYOF, HSD17B12, PRDX2, APCS, PLA2G2A SERPINH1/HSP47 and MVP) could be used as potential markers for chondrocyte differentiation, since they show high difference in abundance levels and are identified with a very high frequency in the sample population that was tested.

## Results

### Identified and differentially synthesized proteins

**Figure 1:**
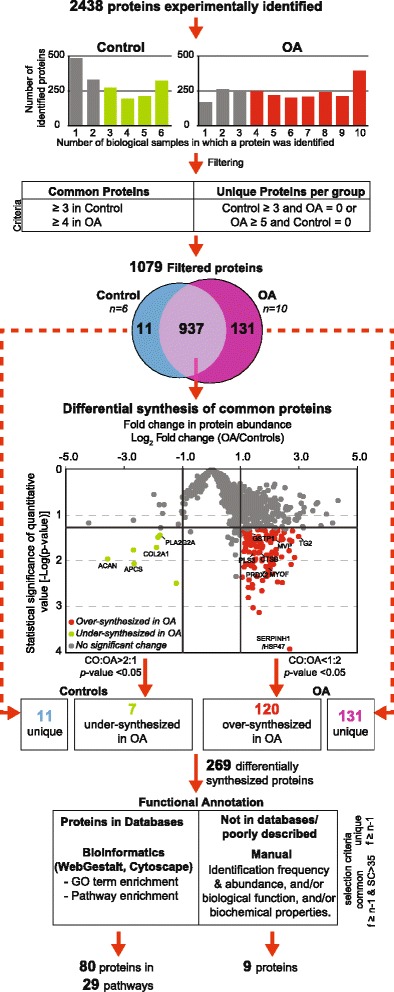
Proteomic data analysis workflow. Experimentally identified proteins were filtered for the identification of differently synthesized proteins (see Methods). Two protein sets (one for controls and one for OA) were then functionally annotated using WebGestalt and ClueGO. In addition, 9 proteins robustly identified but poorly described in databases, were selected.

About 2,400 proteins were identified in total in all samples at least once (see Methods; Additional file 1 – “Total Proteins Identified”). To ensure robustness of identification of proteins as characteristic of one or the other or both study groups we imposed stringent criteria (see Methods). A total of 142 unique and 937 common proteins were thus confidently assigned (Figure 1, Additional file 1 – “Unique IDs in OA”, “Unique IDs in controls”, “Common Proteome-filtered”). None of the peptides of the unique proteins could be identified in the opposite study group. Lack of identification for a given protein indicates that it is either not synthesized at all or that it exists in amounts that are below detection [22] (see below).

**Figure 2:**
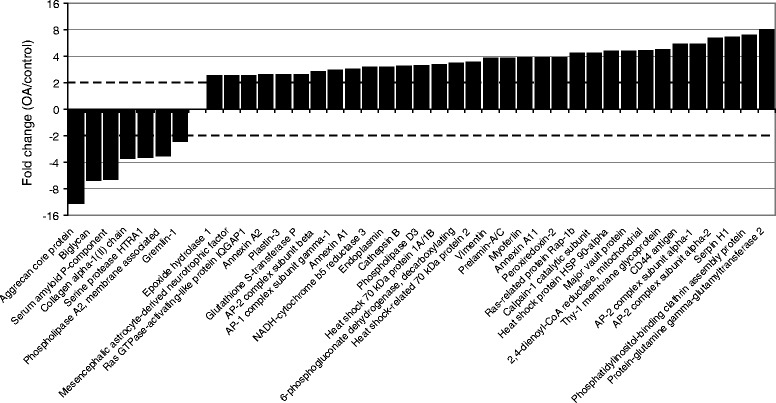
Over-/under-synthezied proteins in OA chondrocytes. Several commonly identified proteins show increased or decreased synthesis in OA chondrocytes, compared to the control group. The complete table with the over- and under-synthesized proteins is shown Additional file 1 – “Common Proteome – Filtered”.

The majority of the identified proteins (~85%) was found in both study groups and presumably reflects the common chondrocyte proteome. Attesting to this, many of the proteins characteristic for chondrocytes, e.g. ACAN, COL2A1 were identified [23]. To determine over- or under-synthesized proteins in the “common” dataset, we compared the quantitative values in the OA and control groups. For this, a spectral counting label-free quantification approach was used in the identified proteins, as implemented in Scaffold software [24]. The abundance of proteins of the “common proteome” was tested for statistical significance by applying a *t-test* [25]. Also, in the same subset the fold change was calculated by dividing the mean quantitative value of the OA group with the mean quantitative value of the control group. Significantly over-synthesized proteins in the OA group are those that have p-values <0.05 and fold change > 2 (or log_2_ fold change > 1), whereas in the control group those that have a p-value <0.05 and fold change < 0.5 (or log_2_ fold change < −1) (Figure 1). Overall, 127 of the proteins shared between the chondrocytes from the OA and control groups, showed differential abundance (42 of them shown in Figure 2; for the complete set see Additional file 1 – “Over-synthesized in OA” and “Under-synthesized in OA”).

**Figure 3:**
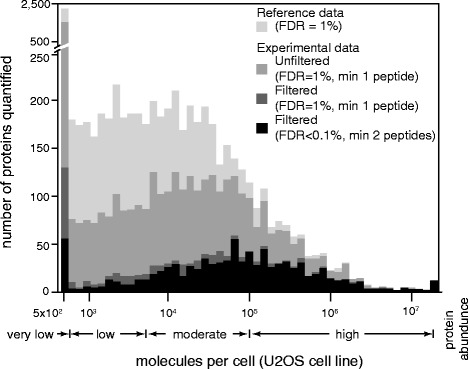
Estimated abundance for the experimentally identified proteins. Experimentally identified proteins were compared with a reference dataset of an osteosarcoma cell line (U2OS cell line) [26]. X-axis: copies per cell, for the proteins identified in the reference proteome; Y-axis: number of proteins correspond to a specific abundance window. We align the experimentally identified proteins with the reference set, and plot the number of proteins from this study that correspond to the reference abundance levels. Light grey histogram: protein abundance distribution of the reference set; mild grey: protein abundance of 4,169 from the 7,367 of experimentally identified proteins using loose criteria (protein FDR = 1% and minimum number of peptides = 1); dark grey: experimentally identified proteins (1% FDR, min 1 peptides) filtered based on the frequency of identification of each protein in the biological samples, using criteria described in Methods (1,194 of 1,475 proteins are plotted); back columns: filtered experimentally identified proteins as described in Figure 1 - venn diagram (947 of the 1,079 proteins are plotted).

Protein copy numbers in a eukaryotic cell show a broad distribution, ranging across at least seven orders of magnitude [26]. Previous proteomics studies have shown similarities in protein levels between cell lines and also certain differences [27,28]. To estimate the lowest protein amounts that can be commonly detected in our study, we compared the abundance of identified proteins to that from a reference study carried out using a human cell-line [26]. Most of the proteins identified here, correspond to those of moderate to high abundance in the reference dataset (Figure 3). However, several other proteins that we identified using strict criteria and filtering based on frequency of identification (see Methods), fell in the low-abundance group of the reference set (Figure 3, black bars). This suggests that our pipeline has a significant identification depth (down to 5x10^2^ molecules/cell). In addition, we see that the filtering step that we include is important for the removal of random identifications. When we analyze our dataset using more loose criteria (protein FDR = 1% and minimum number identified peptides per protein = 1) we can identify ~7,000 proteins in total. If in this dataset we apply filtering based on the frequency of identified samples, we end up with ~1,400 proteins, reducing that way the number of missing values in the dataset.

### Bioinformatic analysis for enriched terms

We next sought to determine biological processes and pathways that may be associated with OA pathogenesis in our experimental dataset. For this, we used *in silico* tools to determine which proteins or biological terms or pathways are enriched in the identified proteins. First, we used WebGestalt [29,30], which performs a hypergeometric test for the enrichment of GO terms and pathways in the selected proteins, followed by the Benjamini & Hochberg (BH) method for multiple test adjustment (adjP). We analyzed the 269 unique or differentially synthesized proteins in our dataset (Figure 1; Additional file 1 – “Unique IDs in OA”, “Over-synthesized in OA”, “Under-synthesized in OA”, “Unique IDs in Controls”), divided in two sets: the first consisted of 120 over-synthesized and 131 unique proteins in OA patients; the second consisted of seven under-synthesized proteins in the patients’ group and 11 unique proteins in the control subjects. Below we present a summary of the results of the WebGestalt analysis.

### Enrichment analysis of GO-terms using WebGestalt

**Figure 4:**
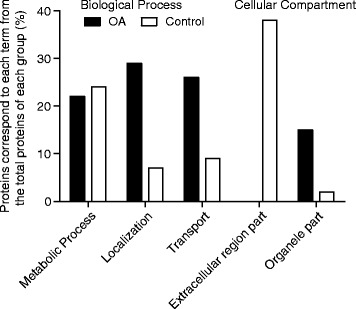
Differentially enriched GO Terms between OA and normal chondrocytes. Differentially synthesized proteins (over-/under-synthesized in OA and uniquely identified proteins) for each group were tested for enrichment in GO terms of biological process and cellular compartment. A high percentage of the proteins belonging to OA group are associated with localization and transport biological functions. The majority of the proteins related to the control group are localized in the extracellular region whereas a higher percentage of the proteins associated with the OA chondrocytes is a part of cellular organelles. Percentages correspond to the number of proteins of each group, associated with a specific term from the total proteins of the specific group. An extended table with the enriched GO terms is provided in Additional file 4.

We identified enriched terms for the categories “biological process” (BP), and “cellular compartment” (CC) of GO between the differentially synthesized proteins of each group (see “Statistical and Enrichment Analysis”, Methods). Enriched GO terms (adjP ≤10^−5^) for each of the BP and CC can be grouped into 10 generic functional categories. Figure 4 illustrates the percentage of proteins that belong to selected generic functional categories from the proteins that are enriched or uniquely identified in each of the study groups (OA or control) (Additional file 2 – Cluster 2 “Protein Set for normal chondrocytes”; Additional file 3 – Cluster 1 “Protein Set for OA chondrocytes”). An extended table with enriched GO terms is included in Additional file 4. Proteins related to metabolic processes, are equally assigned in both study groups, reflecting that common mechanisms of the cell are retained during disease (Figure 4). In OA chondrocytes a high percentage of the differentially synthesized proteins are related to biological processes such as localization and transport mechanisms of the cell, categories that are less represented in control chondrocytes. For the GO category “cellular compartment”, proteins related to the extracellular matrix are significantly under-synthesized in OA chondrocytes (Figure 4). Proteins that form part of cellular organelles are significantly over-synthesized in OA chondrocytes. The remaining groups for BP and CC tested did not show any significant difference between groups (data not shown). These changes clearly highlight that hypertrophic osteoarthritic chondrocytes are phenotypically different from control cells and harbour characteristic proteomic signatures.

### Enrichment analysis of GO-terms using ClueGO

To further probe the GO enrichment analysis presented above, we made use of the ClueGO plug-in of Cytoscape 3.1.0, which enables comparison of clusters/groups to highlight their functional specificity. For this, two protein sets were uploaded in ClueGO (Additional file 2 – Cluster 2 “Protein Set for normal chondrocytes”; Additional file 3 – Cluster 1 “Protein Set for OA chondrocytes”) and were compared to each other by applying appropriate settings (see Methods).

ClueGO was used for the integration of GO terms as well as the generation and visualization of a functionally arranged GO term network that exposes the relations between the GO terms based on the similarity of their associated proteins. ClueGO utilizes kappa statistics, in a similar way as described by Huang et al. [31], to connect the GO terms in the network. Briefly, *kappa* statistics refers to the quantitative measurement of the degree of agreement on how proteins share similar annotation of GO terms.

**Figure 5:**
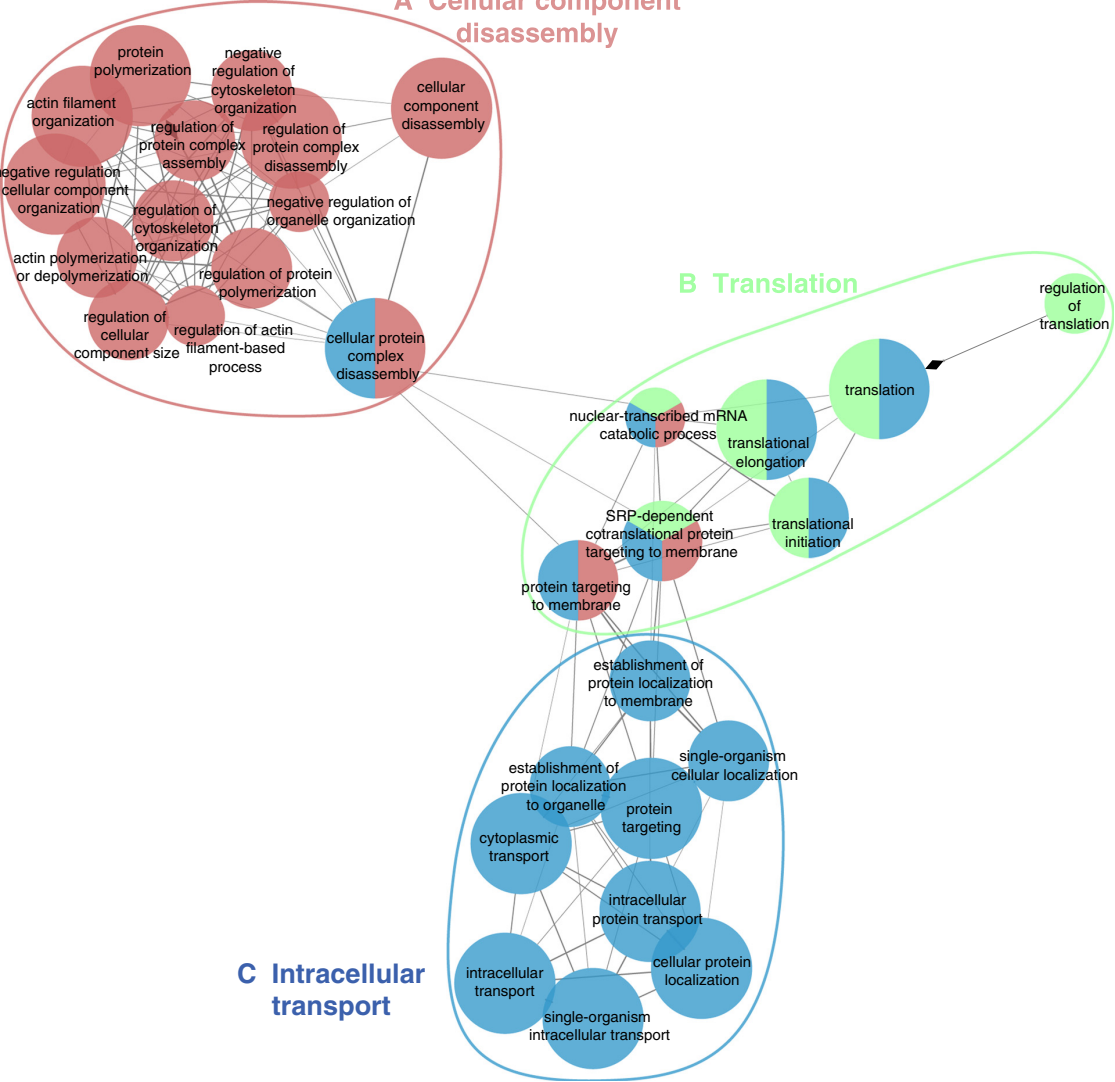
Enriched GO network groups using ClueGO. Biological processes (GO category) of the identified over- and under-synthesized proteins in our experimental dataset were visualized with ClueGO (kappa score ≥ 0.3) as a functional grouped network and only the most significant interactions are shown. Each node represents a biological process. Edges represent connections between the nodes and the length of each edge reflects the relatedness of two processes. The specific functional terms that were used to generate the network in Cytoscape resulted from the comparison of the two clusters (OA and controls; see Methods). The most significant parent or child term per group is shown in the ClueGO grouped network as a group title (**A.** Cellular component disassembly; **B.** Translation; **C.** Intracellular transport). The enrichment significance of the GO terms is reflected by the size of the nodes. Node color, represents the class that they belong. Mixed coloring means that the specific node belongs to multiple classes. Ungrouped terms are not shown.

Three major networks of GO terms related to “biological processes” were highly enriched (Figure 5, Groups A-C). The first group contains terms related with regulation of cytoskeleton. The second group is related to the translation process and the third group is related to intracellular transport. Collectively, these data indicate that three groups of biological processes (cytoskeleton organization, translation and intracellular transport) are altered in OA chondrocytes.

### Pathway enrichment analysis

To examine which biological pathways are apparently altered in the OA chondrocytes, we next performed a pathway analysis. Differentially synthesized proteins in OA chondrocytes were searched against the Wikipathways database, using WebGestalt [29,30]. Enriched pathways were grouped into four categories, based on the biological processes that they participate in (Table 1). The first group contains pathways related with general metabolic processes like proteasome, ribosome, electron transport etc. Even if these pathways do not necessarily pinpoint the precise molecular mechanism of the disease, suggest that OA chondrocytes are metabolically more active, compared to control chondrocytes.

**Table 1:** **Enriched pathways in OA chondrocytes**

**PathwayName**	**Gene Names**	**AdjP-value**
**General metabolism and cellular processes**		
Parkin-Ubiquitin Proteasomal System pathway	TUBA1C PSMD4 HSPA1A PSMD2 PSMD6 HSPA2 PSMC1 TUBB	adjP = 1.47e-07
Translation Factors	EIF3E EIF4A1 EEF1G EEF1A1 EEF2 EIF3B EEF1D	adjP = 2.60e-07
Electron Transport Chain	ATP5B COX6C SLC25A5 UQCRFS1 NDUFB4 ATP5A1	adjP = 0.0001
Cytoplasmic Ribosomal Proteins	RPL8 RPLP0 RPL35A RPL23 RPL10 RPL27	adjP = 8.18e-05
Proteasome Degradation	PSMD4 PSMD2 PSMD6 PSMC1	adjP = 0.0018
TCA Cycle	SUCLA2 IDH3G DLST	adjP = 0.0007
Oxidative phosphorylation	ATP5B NDUFB4 ATP5A1	adjP = 0.0098
**Adhesion, cytoskeleton remodeling, cell-cell & cell-matrix interactions, endocytosis**		
Focal Adhesion	ACTG1 RAC1 RAP1B FLNA VASP MAPK1 PDGFRB CAPN1	adjP = 8.18e-05
Regulation of Actin Cytoskeleton	ACTG1 IQGAP1 RAC1 ENAH GSN PFN1 MAPK1 PDGFRB	adjP = 3.12e-05
Integrin-mediated cell adhesion	RAC1 RAP1B CAPN2 VASP MAPK1 CAPN1	adjP = 0.0001
Synaptic Vesicle Pathway	AP2A2 AP2A1 CLTC AP2B1 NAPA	adjP = 8.74e-05
G13 Signaling Pathway	IQGAP1 RAC1 PFN1	adjP = 0.0034
TOR signaling	RAC1 RRAGC PRKAG1	adjP = 0.0032
Senescence and Autophagy	GSN MMP14 CD44 MAPK1	adjP = 0.0100
**Signaling pathways**		
EGF-EGFR Signaling Pathway	GJA1 IQGAP1 RAC1 HGS AP2A1 AP2B1 MAPK1	adjP = 0.0003
TGF beta Signaling Pathway	RAC1 HGS TRAP1 SPTBN1 SKP1 MAPK1 PRKAR2A	adjP = 0.0001
TNF alpha Signaling Pathway	RAC1 PSMD2 TRAP1 BAX SKP1 HSP90AA1 MAPK1	adjP = 2.36e-05
MAPK signaling pathway	RAC1 RAP1B FLNA MAPK1 PDGFRB	adjP = 0.0058
Wnt Signaling Pathway	GJA1 RAC1 CTNNB1	adjP = 0.0063
Corticotropin-releasing hormone	GJA1 RAP1B GNB2 GNAS CTNNB1 HSP90AA1 MAPK1 GNAI2	adjP = 6.10e-06
Androgen receptor signaling pathway	RAC1 FHL2 FLNA CTNNB1 CARM1	adjP = 0.0007
TSH signaling pathway	RAP1B GNAS MAPK1 GNAI2	adjP = 0.0021
TWEAK Signaling Pathway	RAC1 CTNNB1 MAPK1	adjP = 0.0083
RANKL-RANK Signaling Pathway	RAC1 FHL2 MAPK1	adjP = 0.0121
**Lipid metabolism, eicosanoid metabolism, oxidative stress**		
metapathway biotransformation	CYP20A1 GSTT1 GSTP1 GSTK1 GPX4 CYP1B1 EPHX1	adjP = 0.0003
cytochrome P450	CYP20A1 CYB5R1 CYP1B1 CYB5R3	adjP = 0.0018
Fatty Acid Beta Oxidation	CHKB SLC25A20 DECR1	adjP = 0.0148
Prostaglandin Synthesis and Regulation	ANXA6 ANXA1 ANXA2	adjP = 0.0023
Glutathione metabolism	GSTT1 GPX4	adjP = 0.0150

Differently synthesized proteins in OA chondrocytes were subjected to pathway analysis, by querying the Wikipathways database, using the WebGestalt web tool. Enriched pathways are grouped into four categories based on the biological processes participating. Proteins assigned to each pathway are listed in “gene names” column. AdjP-value corresponds to p-value adjusted by the multiple test adjustment, during enrichment test.

The second group includes pathways related with remodeling of cytoskeleton and endocytosis. Pathways mediating focal adhesion, integrin-mediated cell adhesion, G13 and TOR signaling are used in several biological processes including cell motility and proliferation, cell differentiation and gene expression or cell survival and cell cycle regulation, often linking extracellular signals with other intracellular signaling pathways. Several intracellular signaling pathways were also identified in pathway analysis and are listed in the third group. Proteins belonging to EGF-EGFR, TGF-beta, TNF-alpha, MAPK, Wnt, CRH, androgen receptor, senescence and autophagy and TSH signaling pathways showed altered synthesis in hypertrophic chondrocytes. Signaling through these pathways has been described in the literature during normal chondrocyte differentiation or development of the OA disease [32-39]. Two more signaling pathways, TWEAK and RANKL-RANK signaling pathways, which are associated with the production of pro-inflammatory cytokines and bone development were also enriched in OA chondrocytes [40,41].

The fourth group of enriched pathways includes those related to lipid metabolism and oxidative stress. Proteins participating in fatty acid beta oxidation and enzymes of the cytochrome p450, apart from their role in the metabolism of lipids and chemical compounds, are also used for the metabolism of eicosanoids and other secondary metabolites [42].

### Proteins with synthesis characteristic to each group

We next searched for proteins that show distinct synthesis in each group, and potentially underscore the phenotypic shift in OA chondrocytes. Most *in silico* function annotation tools use public pathway databases to assign the identified proteins/genes to specific pathways. However, several proteins are not included in these databases, mainly because their interactions are not completely defined, and cannot be functionally annotated by the pathway enrichment tools. In our dataset, there were a few such proteins, which show distinct synthesis patterns between OA and normal chondrocytes, and are not included in the *in silico* pathway analysis described above, or their protein function is not completely described by the pathway analysis.

**Figure 6:**
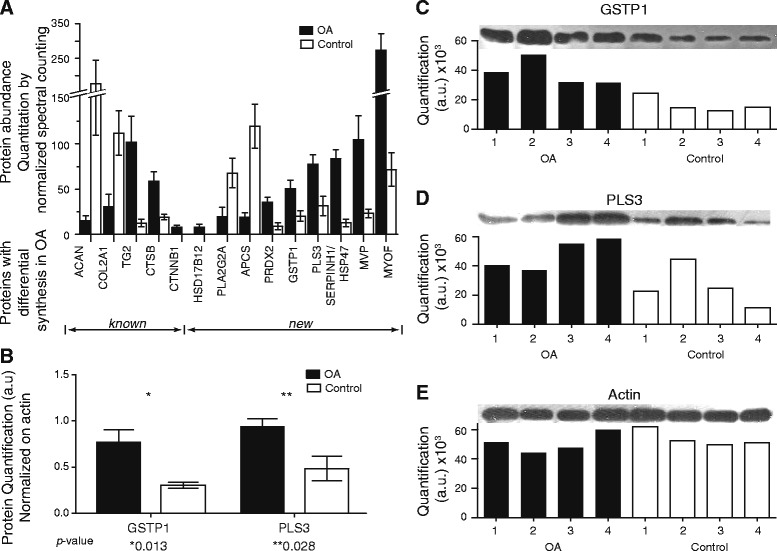
Differentially synthesized proteins. **A)** Proteins with different abundance levels between the two groups, as determined by label-free quantification after LC-MS. Quantitative values shown correspond to average values between the total samples in which a given protein was identified (ACAN: OA = 9, control = 6; COL2A1: OA = 5, control = 5; TG2: OA = 10, control = 6; CTSB: OA = 10, control = 6; CTNNB1: OA = 9, control = 0; HSD17B12: OA = 9, control = 0; PLA2G2A: OA = 5, control = 5; APCS: OA = 4, control = 5; PRDX2: OA = 10, control = 6; GSTP1: OA = 10, control = 6; PLS3: OA = 10, control = 5; SERPINH1/HSP47: OA = 9, control 6; MVP: OA = 10, control = 6; MYOF: OA = 10, control = 6). Error-bars correspond to ±1 s.e. **B)** Western blot analysis for the validation of label-free quantication method, using antibodies against GSTP1 and PLS3 in four different OA and control samples. Values are normalized to β-actin. Error-bars correspond to ±1 s.e. **C)** Quantitation (arbitrary units) of GSTP1 per sample. **D)** Quantitation (arbitrary units) of PLS3 per sample **E)** Quantitation (arbitrary units) of β-actin per sample.

We selected proteins that are not previously described in OA chondrocytes and are among the most confidently identified proteins (show very high frequency of identification between all samples and have high number of spectra in the label-free quantification). We ended up with nine proteins (HSD17B12, PLA2G2A, APCS, PRDX2, GSTP1, PLS3, SERPINH1, MVP, MYOF), described in Figure 6A. Eight of them are differentially synthesized between OA and normal chondrocytes whereas HSD17B12 is identified only in OA chondrocytes with very high frequency (9 out of 10). We also selected several control proteins for the validation of the quantification method (ACAN, COL2A1, TG2, CTSB, CTNNB1) (Figure 6A). All of the control proteins showed similar synthesis pattern as expected [35,43-45].

To corroborate the label-free quantification approach, we performed western blot analysis for two of the selected proteins (GSTP1 and PLS3), in four new samples of OA chondrocytes and equal number of controls (Figure 6B-E). For loading control and normalization between samples we probed for β-actin. Western blot analysis was in agreement with the mass spectrometric label-free quantification. The two proteins were found in 2.5 and 2 fold higher abundance in OA patients than in the controls, accordingly.

## Discussion

To probe the multifactorial nature of osteoarthritis, we used an –omics approach that offers a broad, unbiased view of the OA landscape, in order to improve our understanding on the molecular base of the disease. We performed a quantitative comparative proteomic analysis, of osteoarthritic and normal chondrocytes, combined with bioinformatics analysis.

During OA progression, chondrocytes acquire a hypertrophic phenotype, characterized by altered synthesis of many proteins related to normal development and cartilage turnover. Hypertrophy is a physiological stage in chondrocyte differentiation, and an essential step during bone development [17,18,46,47]. In this study, we showed that hypertrophic osteoarthritic chondrocytes possess a distinct synthesized proteome compared to that of the control group. Major biological processes that are affected during disease progression, are related to the cytoskeleton, cell-cell and cell-ECM interactions. In agreement with this, several proteins over-synthesized in OA chondrocytes are related to cell localization and transport (Figure 4). Network analysis for GO terms, using ClueGO (Figure 5), showed that a significant number of the differentially synthesized proteins cluster together having biological functions related to the cytoskeleton, whereas several pathways, such as regulation of actin cytoskeleton, integrin-mediated adhesion and the G13 and TOR signaling pathways where found enriched in OA chondrocytes (Table 1). The interaction of chondrocytes with ECM molecules has been shown to affect cytoskeletal organization, proliferation, differentiation, and gene expression in chondrocytes [48-54]. Also, it is noted that the micro-environment of cartilage possibly triggers an autophagy response in the hypoxic chondrocytes [55]. Collectively, these data demonstrate a global view of cytoskeleton-related changes associated with OA chondrocytes.

Several signaling pathways are activated during chondrocyte differentiation [16,46]. Apart from cytoskeleton-related pathways, we also found that other signaling pathways are enriched in OA chondrocytes. These include EGF-EGFR, TGF-beta, TNF-alpha, MAPK and Wnt canonical and non-canonical signaling pathways, TWEAK and RANKL-RANK signaling pathways (Table 1). Signaling through those pathways has been reported to occur during normal chondrocyte differentiation or OA development in arthritis models, whereas the RANKL-RANK pathway was also proposed as a target for treatment of OA [16,32-35,40,41,46,56-58].

In addition, pathway enrichment analysis showed that signaling through corticotrophin releasing hormone (CRH), androgen receptor (AR) and thryroid stimulating hormone (TSH) is also affected in OA chondrocytes. CRH signaling plays a role in inflammatory responses and its enrichment could possibly reflect joint inflammation [37]. Expression of AR in articular chondrocytes was assessed in human and mouse biopsies. The percentage of cells showing increased expression of AR is increased in resting and hypertrophic zones compared to the proliferative zone, suggesting that signaling through AR might be used during normal chondrocyte differentiation [36,59]. Also, differentiation of mesenchymal stem cells into chondrocyte-like cells was observed after treatment with TSH [60]. However, the authors conclude that further studies are needed in order to determine the exact role of TSH in the chondrocyte differentiation process and whether this pathway may promote osteogenic differentiation, as well.

Another group of pathways that is enriched in OA chondrocytes includes those related to lipid metabolism, prostaglandin synthesis, glutathione metabolism and metabolism through the cytochrome p450 (Table 1). Enzymes of those pathways are also used for the metabolism of eicosanoids [42]. Several secondary metabolites, including prostaglandins (PG), epoxyeicosanoic acids (EET), hydroxyeicosatetranoic acids (HETE) are synthesized from eicosanoids, and regulate a plethora of biological functions, including inflammation, cell growth, survival, migration and invasion [42,61-66]. Altered eicosanoid metabolism was reported before in OA articular chondrocytes [21]. Elevated synthesis of proteins of glutathione metabolism could be induced by increased oxidative stress in the OA cells, a finding that is corroborated by another proteomic study [20]. In addition, we found several proteins, which are related with previously described biological processes, but which have not yet been related to OA or shown to be over-synthesized in OA chondrocytes. These include mainly GSTP1 and HSD17B12 (Figure 6A). The levels of GSTP1 protein were also verified using WB (Figure 6B). GSTP1 is important for metabolite detoxification [67]. Over-expression of the GSTP1 gene was observed in several cancer types and is considered to be a marker for cancer development [68]. Additionally, GSTP interacts directly and can inhibit c-Jun N-terminal kinase (JNK), affecting that way processes like apoptosis, differentiation and proliferation [68-70]. The expression of HSD17B12 was associated with the metastatic phenotype in tumor cell lines, through its function in Arachinoid Acid (AA) metabolism, linking the metabolism of eicosanoids and cytoskeleton remodeling [71,72]. Thus, activation of lipid metabolism pathways, might lead to altered production of secondary metabolites which in turn support cytoskeleton reorganization during synovial inflammation and chondrocyte differentiation, phenotypes well defined during OA.

Two more proteins which are over-synthesized in OA chondrocytes, and are associated with the observed phenotype are PLS3 and MYOF. PLS3 synthesis levels were also verified with WB (Figure 6B). Pathogenic variants of this protein were found in families with X-linked osteoporosis and fractures, suggesting that PLS3 might be important in human bone health [73]. The synthesis levels and function of PLS3 has not been studied in chondrocytes. Over-synthesis of PLS3 was found in cancer cells and is proposed as a marker of circulating tumor cells (CTCs) [74]. The exact function of PLS3 is not defined. However, it seems to function as a key downstream molecule in the TGF-β pathway [75]. In addition, we observed significantly increased synthesis of MYOF in OA chondrocytes, a protein that is related to membrane repair by an unknown mechanism [76,77]. Over-synthesis of MYOF in breast cancer cell lines leads to an increased invasion phenotype and secretion of matrix metalloproteinases (MMPs), while silencing of the gene restores the normal phenotype, possibly by blocking the EGFR signaling pathway [78,79]. Both proteins seem to have a key functional role, since silencing of their genes can reverse the phenotype induced by the initial stimuli.

Additionally, we identified five more proteins with distinct synthesis between OA and control chondrocytes, and for which we do not have any knowledge about the function in relation to the disease. From these proteins PRDX2, SERPINH1/HSP47 and MVP are over-synthesized in OA chondrocytes, whereas APCS and PLA2G2A are mainly synthesized in control chondrocytes (Figure 6A). Peroxiredoxin-2 (PRDX2) is a member of a class of thiol peroxidases and is hypothesized to act as a redox sensor [80]. SERPINH1/HSP47 is a collagen specific molecular chaperone, essential for the maturation of type I and type III collagens [81]. Major vault protein (MVP) is an evolutionally conserved, high abundant protein that seems to play a role in drug resistance and in signaling pathways, however its role is not understood [82,83]. Serum amyloid P component (APCS) is a member of pentraxin family of proteins, that plays a role in innate immune responses [84]. Its presence in cartilage was previously reported, however nothing is known about the role of this protein in OA [85]. Finally, the membrane-associated form of phospholipase A2 (PLA2G2A), is also highly synthesized in control chondrocytes compared to the OA, but the function of this protein is unknown [86].

## Conclusion

In summary, we observed that OA chondrocytes display molecular signatures of the hypertrophic phenotype, where several pathways including adhesion, proliferation, growth, and lipid metabolism are enriched. We propose that the expression of the proteins GSTP1, PLS3, MYOF, HSD17B12, PRDX2, APCS, PLA2G2A SERPINH1/HSP47 and MVP also reflect the altered phenotype of OA chondrocytes. Our study provides a number of testable hypotheses to further probe the role of various proteins and biological process pathways in osteoarthritis.

## Methods

### Cartilage tissue samples

Articular cartilage samples were obtained from femoral heads, femoral condyles and tibial plateaus of patients with primary osteoarthritis undergoing knee replacement surgery at the Orthopaedics Department of the University Hospital of Larissa [87]. Ten OA patients who had undergone total knee replacement surgery were included. Each articular cartilage sample was categorized according to its gross morphology as severely damaged and was taken from the main defective area of maximal load. Kellgren-Lawrence grading scale (K-L) was used for the assessment of OA severity in OA and control samples (Table 2) [88]. Patients with rheumatoid arthritis and other autoimmune disease as well as chondrodysplasias, infection-induced OA and post-traumatic OA were excluded from the study. Normal cartilage was obtained from six individuals undergoing fracture repair surgery, with no history of joint disease. Both patients and healthy individuals’ cartilage samples were obtained upon verbal informed consent form all individuals. The method of obtaining verbal consent was approved by the Institutional Review Board of the University Hospital of Larissa. The study protocol conformed to the ethical guidelines of the 1975 Declaration of Helsinki as reflected in *a priori* approval by the Local Ethical Committee of the University Hospital of Larissa.

**Table 2:** **Samples information**

**Sample**	**Group**	**Sex**	**K-L score**
332OA	OA	F	4
393OA	OA	F	4
520 ΟΑ	OA	M	4
524OA	OA	F	3
525OA	OA	F	4
526OA	OA	F	4
529OA	OA	F	4
530OA	OA	F	4
532OA	OA	F	4
533OA	OA	F	4
150Α	Controls	F	1
151Α	Controls	M	0
152Α	Controls	M	0
153Α	Controls	F	0
154Α	Controls	M	1
175Α	Controls	F	0

10 OA and 6 control chondrocyte samples were studied. OA stage was measured in Kellgren & Lawrence system (K-L).

### Primary cultures of human articular chondrocytes, normal and osteoarthritic

Articular cartilage was transported from the surgical room to the laboratory in HBSS medium (Hanks Balanced Salt Solution). It was then immediately dissected and subjected to sequential digestion with pronase (1 mg/ml; 90 min; Roche Applied Science, Germany) and collagenase P (1 mg/ml; 3 h; Roche Applied Science, Germany) at 37°C. Chondrocytes were cultured in Dulbecco’s Modified Eagles Medium/ Ham’s F-12 (DMEM/F-12) (GIBCO BRL, UK) plus 5% fetal bovine serum (FBS) and 100 U/ml penicillin-streptomycin and were incubated at 37°C under a humidified 5% CO_2_ atmosphere. Chondrocytes were kept in culture for maximum of two passages. Trypsin was used for the detachment of chondrocytes during the primary culture, while type II collagen and type I collagen ratio was screened in all samples to exclude dedifferentiation events.

### Protein extraction from chondrocytes

OA and normal chondrocytes were trypsinized, collected and centrifuged for 10 min at 543xg. The cell pellet was washed with PBS and then centrifuged again for 10 min at 543xg at 4°C. The cell pellet was resuspended in RIPA lysis buffer (50 mM Tris/HCl pH 7.2, 150 mM NaCl, 1% Triton X-100, 1% sodium deoxycholate, 0.1% SDS) and incubated on ice for 30 min. Remaining cells where lysed after sonication (3 rounds at 30% amplitute), using a tip sonicator (VCX130, Sonics & Materials, Newtown, USA). Samples were clarified via centrifugation (15 min; 13,000xg; 4°C), in a bench top centrifuge, and total protein content was calculated using the BCA method [89].

### 1D-SDS-PAGE and in-gel digestion

Proteins from each sample (50 μg) were precipitated using trichloroacetic acid/acetone (final concentration of 25% TCA w/v; 4°C; 30 min). Proteins were precipitated after centrifugation (15,000 g; 30 min; 4°C), in a bench-top centrifuge. Protein pellets were washed twice with ice-cold acetone, and precipitated via centrifugation, as previously. Excess acetone was aspirated and the protein pellet was air-dried. Precipitated proteins were re-solubilized in a solution containing 1.5 M Tris/HCl pH 8.8 and 6x sample buffer in a ratio of 2:1 and analyzed by 1D-SDS-PAGE 4-10% (29:1 acrylamide/bisacrylamide). Gels were stained with colloidal coomassie blue (Coomassie G250, 10% phosphoric acid, 10% ammonium sulfate, 20% methanol). Each lane was cut in 10 slices, and de-stained after three washes with 50% acetonitrile/water and 50 mM ammonium bicarbonate. Samples were reduced in the presence of 10 mM DTT (45 min; 56°C) and then washed and alkylated in the presence of 55 mM IAA (45 min; 22oC, shaking, in the dark). Gel slices were washed with 50 mM ABS and proteins were digested with 0.1 μg trypsin (trypsin Gold, Promega, Fitchburg, Wisconsin), overnight. Generated peptides were collected, after repeated washes with nanopure-H_2_O, 50% ACN in low binding tubes (Axygen, Union City, CA) and trypsin was quenched by acidifying the sample with 1–2 μL trifluoroacetic acid (TFA), until pH < 2. Peptides were dried under vacuum, during centrifugation (Speedvac; Savant) and desalted using STAGE tips [90]. The desalted peptides were then lyophilized (Speedvac) and stored at −20°C until use.

### LC-MS/MS analysis

Peptide samples were analyzed using nano-Reverse Phase (RP) LC coupled to an LTQ-Orbitrap XL or an Orbitrap QE instrument. In the first case, peptides were separated by an EASY-nLC 1000 HPLC (Proxeon, Thermo Fisher Scientific, Odense, Denmark) system on a pre-packed column (Thermo, OD 360 μm, ID 50 μm, 15 cm, C18, 2 μm). A linear gradient was used 5-30% from buffer A (99.9% water, 0.1% FA) to buffer B (9.9% water, 0.1% FA, 90% ACN) run for 165 min (flow rate: 300 nL/min), and peptides were analyzed in an LTQ-Orbitrap XL, at a resolution of 60,000 (FWHM) at m/z 400. CID fragmentation was performed on the 10 most intense precursor ions using 60 seconds exclusion time. For the analysis in the Orbitrap QE mass spectrometer, peptides were separated in a Dionex UltiMate 3000 UHPLC system. The samples were separated using an EasySpray C18 column (Thermo Scientific) in a linear gradient using as buffer A (water 99.9%, FA 0.1%) and buffer B (ACN 80%, water 20%, FA 0.1%). Gradient from buffer A to buffer B was separated in five steps, 4% to 10% B (12 min) followed by 10-35% B (20 minutes), 35- 65% B (5 min) and a final elution and re-equilibration step at 95% and 5% B respectively. The flow-rate was set at 300 nL/min. The Q Exactive was operated in positive ion mode (nanospray voltage 1.5 kV, source temperature 250°C). The instrument was operated in data-dependent acquisition (DDA) mode with a survey MS scan at a resolution of 70,000 for the mass range of m/z 400–1600 for precursor ions, followed by MS/MS scans of the top 10 most intense peaks with +2, +3 and +4 charged ions above a threshold ion count of 16,000 at 35,000 resolution using normalized collision energy (NCE) of 25 eV with an isolation window of 3.0 m/z, an apex trigger 5–15 sec and a dynamic exclusion of 10 s. All data were acquired with Xcalibur 2.2 software (Thermo Scientific).

### MS data analysis

Raw files were processed in Proteome Discoverer v1.4 using the algorithms SEQUEST v1.1.0.263 and Mascot v2.3.2. Spectra were searched against the Uniprot Human reference database, without isoforms (Oct 2012, 20,505 entries) [91]. Precursor mass error was set to 10 ppm for both Orbitrap mass spectrometers, and fragment mass error to 800 mmu or 20 mmu, for the LTQ Orbitrap XL and Orbitrap QE, respectively. Enzyme cleavage was set to trypsin, and maximally two missed cleavages were allowed. Variable modifications selected were methionine oxidation and N-terminal acetylation; constant modification selected was S-Carbamidomethylation of cysteine residues. All files were merged in Scaffold v3.4.5 (Proteome Software, Portland, OR) for the comparative analysis. Scores from both Mascot and SEQUEST algorithms was used through the PeptideProphet and ProteinProphet algorithms for the identification of proteins [92-94]. Thresholds for protein and peptide identification through ProteinProphet and PeptideProphet algorithms were set to 99% and 95% accordingly, for proteins with minimum 2 different peptides identified, resulting in a protein false discovery rate (FDR) of <0.1%. For the label-free quantification of the identified proteins, we used spectral counting quantification through Scaffold software, which sums all the spectra associated with a specific protein within the sample and also includes these spectra that are shared with other proteins [95].

### Statistical and enrichment analyses

Protein lists were exported from Scaffold to Microsoft Excel for further processing. To ensure robustness of identification, we filtered-out proteins identified with a low frequency. Specifically, we accepted as “uniquely identified” proteins those, which were identified in at least half of the samples of each group (n ≥ 3 in the control group and n ≥ 5 in OA group). From the commonly identified proteins between OA and control conditions, we discarded proteins with a frequency of identification < 4 and <3, accordingly.

For the identification of differentially synthesized proteins, we tested for statistical (*t-test*) and biological (fold-change) significance. A two tail *t-test* was performed in the filtered “common sub-proteome” described before (n ≥ 3 in the control group and n ≥ 4 in OA group) [25]. Missing values were excluded. Also for the same sub-proteome, the fold change of protein levels was calculated by dividing the mean spectral counting quantitative value in OA samples with the mean value of the control samples for each of the proteins. Proteins that were significantly “over-synthesized” or “under-synthesized” were these with p-value ≤0.05 and a fold change ≥ 2.

For the enrichment analysis we divided these proteins in two groups. The first group contains proteins that are over-synthesized and uniquely identified in OA chondrocytes and the second group contains proteins which are under-synthesized in OA chondrocytes, compared to control samples, or were uniquely identified in control chondrocytes. The two protein groups were submitted separately to WebGestalt as lists of their respective Entrez gene IDs in order to enable a distinct enrichment analysis after comparison with the existing list of the human genome, which is generated from prior knowledge sorted into functionally related gene/protein groups. WebGestalt performs the hypergeometric test for the enrichment of GO terms and pathways in the selected proteins, followed by the Benjamini & Hochberg (BH) method for multiple test adjustment (adjP) [29,30]. As we are testing multiple gene sets at the same time, the p-values need to be adjusted. BH is implemented to control for false positive (i.e. Type I) errors. Traditional multiple-testing corrections, adjust p-values derived from multiple statistical tests to correct for the occurrence of false positives. BH ranks p-values in an ascending order, multiplies them by the number of features, and divides them by their corresponding rank [96]. Using WebGestalt, we conducted a functional enrichment analysis for Gene Ontology (GO) terms, as well as a pathway enrichment analysis using the Wikipathways database (http://www.wikipathways.org).

### Cluster/Group comparison by ClueGO

ClueGO, a Cytoscape plug-in was used to visualize the non-redundant GO terms and pathways in functionally organized networks, which reflect the relations between the biological terms based on the similarity of their linked gene/proteins [97]. For the cluster/group comparison by ClueGO, we used the two protein groups, as mentioned above, in order to illustrate their functional differences. Using the Cytoscape environment [98], the two protein groups were uploaded in ClueGO as two separate clusters (Additional file 2 – Cluster 2 “Protein Set for normal chondrocytes”; Additional file 3 – Cluster 1 “Protein Set for OA chondrocytes”) from the text files, and were compared with each other by applying the following settings.

For the enrichment of biological terms and groups, we used the two-sided (Enrichment/Depletion) tests based on the hyper-geometric distribution. We set the statistical significance to 0.05 (p ≤ 0.05), and we used the Bonferroni adjustment to correct the p-value for the terms and the groups created by ClueGO. We used fusion criteria to diminish the redundancy of the terms shared by similar associated proteins, which allows one to maintain the most representative parent or child term. The Kappa-statistics score threshold was set to 0.3. Other analysis parameters include: GO level intervals: (3–15); number of associated proteins for cluster 1: (nine); number of associated proteins for cluster 2: (one); OR 50% specific; GO Term Fusion; Leading Group: Highest Significance; Group Merge: (50%).

### Western blot analysis

Chondrocytes were lysed as previously described and protein concentration was quantified using the Bradford protein assay (Bio-Rad Protein Assay, BioRad, Hercules, CA) with bovine serum albumin as standard. Cell lysates from chondrocytes were electrophoresed and separated on a 10% acrylamide gels and transferred to PVDF membranes (Millipore) that were probed with polyclonal rabbit anti-GSTP1 and anti-PLS3 antibodies (Sigma-Aldrich, Missouri, USA). Polyclonal rabbit Anti-β-actin antibody was used as loading control (Sigma-Aldrich, Missouri, USA). Signals were detected using suitable immunoglobulin IgG conjugated with horseradish peroxidase. Nitrocellulose membranes were exposed to photographic film, which was scanned and intensities of protein bands were determined using ImageJ software.

### Other software

Images and vectors were processed using Adobe Illustrator CS5 and Graphs were designed in GraphPad Prism.

## Additional files

Additional file 1:
**Identified Proteins in OA and control chondrocytes.** Description of data: Total proteins identified in this study and protein sub-groups based on the quantitative value and frequency of identification (see Methods). Additional file 2:
**Cluster 2 - Protein Set for normal chondrocytes.** Under-synthesized proteins in OA chondrocytes and Uniquely identified proteins in normal chondrocytes. Additional file 3:
**Cluster 1 - Protein Set for OA chondrocytes.** Over-synthesized in OA chondrocytes and Uniquely identified proteins in OA chondrocytes. Additional file 4:
**Enriched GO terms in differentially synthesized proteins.** Summary of detailed GO terms enriched in OA and control chondrocytes, after GO enrichment analysis using WebGestalt.

## Abbreviations

AA: Arachidonic Acid
ABS: Ammonium bicarbonate solution
ACAN: Aggrecan core protein
ACN: Acetonitrile
APCS: Serum amyloid P-component
AR: Androgen receptor
BP: Biological process
CC: Cellular compartment
CID: Collision induced dissociation
COL2A1: Collagen type II alpha 1 chain
CRH: Corticotropin releasing hormone
CTNNB1: Catenin beta-1
CTSB: Cathepsin B
DDA: Data dependent acquisition
EPHX1: Epoxide hydrolase 1
FDR: False discovery rate
GO: Gene Ontology
GSTP1: Glutathione S-transferase P1
HSD17B12: Estradiol 17-beta-dehydrogenase 12
IAA: Iodoacetic acid
K-L: Kellgren-Lawrence grading scale
MF: Molecular function
MVP: Major vault protein
MYOF: Myoferlin
NCE: Normalized collision energy
OA: Osteoarthritis/Osteoarthritic
PLA3G2A: Phospholipase A2, membrane associated
PLS3: Plastin-3
PRDX2: Peroxiredoxin-2
RP: Reverse phase
SC: Spectral counting
TFA: Trifluoroacetic acid
TG2: Transglutaminase-2
TSH: Thyroid stimulating hormone
