# Supplementary material for: Comparative proteomic analysis of hypertrophic chondrocytes in osteoarthritis
Source: Clin Proteomics. 2015 Apr 25;12(1):12. doi: 10.1186/s12014-015-9085-6 (PMC4415313; doi:10.1186/s12014-015-9085-6)
Supplement: Additional file 2: — Cluster 2 - Protein Set for normal chondrocytes. Under-synthesized proteins in OA chondrocytes and Uniquely identified proteins in normal chondrocytes. [file 12014_2015_9085_MOESM2_ESM.pdf]

CLUSTER 2 - Protein Set for normal chondrocytes			
	Uniprot	Gene Symbol	Enrez Gene ID
**Proteins "Under-represented" in OA chondrocytes**	P16112	ACAN	176
	P21810	BGN	633
	P02743	APCS	325
	P02458	COL2A1	1280
	Q92743	HTRA1	5654
	P14555	PLA2G2A	5320
	O60565	GREM1	26585
**Proteins Uniquely identified in Controls**	P02649	APOE	348
	P55107	GDF10	2662
	P01009	SERPINA1	5265
	Q6UXX5	ITIH6	347365
	P01024	C3	718
	Q9UKU9	ANGPTL2	23452
	Q16674	MIA	8190
	Q9NRR1	CYTL1	54360
	P34096	RNASE4	6038
	P02741	CRP	1401
	Q9UQ26	RIMS2	9699
