# Supplementary material for: Comparative proteomic analysis of hypertrophic chondrocytes in osteoarthritis
Source: Clin Proteomics. 2015 Apr 25;12(1):12. doi: 10.1186/s12014-015-9085-6 (PMC4415313; doi:10.1186/s12014-015-9085-6)
Supplement: Additional file 3: — Cluster 1 - Protein Set for OA chondrocytes. Over-synthesized in OA chondrocytes and Uniquely identified proteins in OA chondrocytes. [file 12014_2015_9085_MOESM3_ESM.pdf]

CLUSTER 1 - Protein Set for OA chondrocytes			
	Uniprot	Gene Symbol	Enrez Gene ID
Proteins "Over-represented" in OA chondrocytes	P21980	TGM2	7052
	O94925	GLS	2744
	P50454	SERPINH1	871
	P16070	CD44	960
	P33527	ABCC1	4363
	P04216	THY1	7070
	Q9UM22	EPDR1	54749
	Q16698	DECR1	1666
	Q14764	MVP	9961
	P07900	HSP90AA1	3320
	Q9UBS4	DNAJB11	51726
	P32119	PRDX2	7001
	P50995	ANXA11	311
	Q9NZM1	MYOF	26509
	P02545	LMNA	4000
	P08670	VIM	7431
	Q00325	SLC25A3	5250
	P54652	HSPA2	3306
	Q99623	PHB2	11331
	P58546	MTPN	136319
	Q9NQC3	RTN4	57142
	P08107	*HSPA1B*	3304
	Q15067	ACOX1	51
	Q8IV08	PLD3	23646
	P14625	HSP90B1	7184
	P04083	ANXA1	301
	Q9H8Y8	GORASP2	26003
	Q6S8J3	POTEE	445582
	P50990	CCT8	10694
	P28482	MAPK1	5594
	Q9BUT1	BDH2	56898
	P09211	GSTP1	2950
	P13797	PLS3	5358
	P07355	ANXA2	302
	P55145	MANF	7873
	P07099	EPHX1	2052
	P09382	LGALS1	3956
	Q16658	FSCN1	6624
	P08133	ANXA6	309
	P05141	SLC25A5	292
	Q13492	PICALM	8301
	Q8WX93	PALLD	23022

Proteins "Over-represented" in OA chondrocytes	P35579	MYH9	4627
	O94973	AP2A2	161
	O43592	XPOT	11260
	Q9NVA2	SEPT11	55752
	P55072	VCP	7415
	O95782	AP2A1	160
	P06576	ATP5B	506
	P07437	TUBB	203068
	P68104	EEF1A1	1915
	P17655	CAPN2	824
	P25705	ATP5A1	498
	O60701	UGDH	7358
	P07384	CAPN1	823
	P61224	RAP1B	5908
	Q15008	PSMD6	9861
	Q7Z4H8	KDELC2	143888
	P49419	ALDH7A1	501
	Q9Y6C9	MTCH2	23788
	P29144	TPP2	7174
	P05388	RPLP0	6175
	P60842	EIF4A1	1973
	P05023	ATP1A1	476
	P63000	RAC1	5879
	Q10567	AP1B1	162
	P54920	NAPA	8775
	P04899	GNAI2	2771
	P31943	HNRNPH1	3187
	O43759	SYNGR1	9145
	Q9BQE3	TUBA1C	84790
	Q9HAV0	GNB4	59345
	Q9BSJ8	ESYT1	23344
	Q01082	SPTBN1	6711
	Q13813	SPTAN1	6709
	Q9Y6N5	SQRDL	58472
	P52209	PGD	5226
	P06396	GSN	2934
	Q00610	CLTC	1213
	Q01995	TAGLN	6876
	P47985	UQCRFS1	7386
	P55884	EIF3B	8662
	P07858	CTSB	1508
	P0C0S8	HIST1H2AG	8969
	P00387	CYB5R3	1727
	P53801	PTTG1IP	754
	Q92900	UPF1	5976

**Proteins "Over-represented" in OA chondrocytes**			
	P36957	DLST	1743
	O75323	GBAS	2631
	P13804	ETFA	2108
	P13639	EEF2	1938
	P26641	EEF1G	1937
	Q15019	SEPT2	4735
	P40121	CAPG	822
	O43747	AP1G1	164
	P07737	PFN1	5216
	P27348	YWHAQ	10971
	P47756	CAPZB	832
	P63010	AP2B1	163
	Q07065	CKAP4	10970
	Q8NBJ7	SUMF2	25870
	Q32P44	EML3	256364
	P63261	ACTG1	71
	Q8N8S7	ENAH	55740
	O60763	USO1	8615
	P62879	GNB2	2783
	P46940	IQGAP1	8826
	Q13200	PSMD2	5708
	P63092	GNAS	2778
	P61619	SEC61A1	29927
	P68032	ACTC1	70
	P54709	ATP1B3	483
	Q96IU4	ABHD14B	84836
	O00203	AP3B1	8546
	P13674	P4HA1	5033
	P63104	YWHAZ	7534
	P21333	FLNA	2316
	P10619	CTSA	5476
	Q9H0E2	TOLLIP	54472
	P30040	ERP29	10961
**Proteins Uniquely identified in OA chondrocytes**	P40227	CCT6A	908
	Q53GQ0	HSD17B12	51144
	P35222	CTNNB1	1499
	O15067	PFAS	5198
	Q9NV96	TMEM30A	55754
	Q13620	CUL4B	8450
	O75396	SEC22B	9554
	Q10472	GALNT1	2589
	O43684	BUB3	9184
	Q16678	CYP1B1	1545
	P78344	EIF4G2	1982
	P18077	RPL35A	6165

Proteins Uniquely identified in OA chondrocytes			
	O95433	AHSA1	10598
	P09619	PDGFRB	5159
	P41252	IARS	3376
	O43795	MYO1B	4430
	P61163	ACTR1A	10121
	Q8NBX0	SCCPDH	51097
	Q7L2H7	EIF3M	10480
	Q07812	BAX	581
	Q12797	ASPH	444
	O43772	SLC25A20	788
	P43353	ALDH3B1	221
	O43865	AHCYL1	10768
	O95486	SEC24A	10802
	Q9UM54	MYO6	4646
	P13861	PRKAR2A	5576
	P61086	UBE2K	3093
	Q99829	CPNE1	8904
	Q9Y3Q3	TMED3	23423
	Q9Y285	FARSA	2193
	Q6DKJ4	NXN	64359
	Q9NVI7	ATAD3A	55210
	P36969	GPX4	2879
	Q8WWI5	SLC44A1	23446
	P52788	SMS	6611
	Q9NRY6	PLSCR3	57048
	Q93077	HIST1H2AC	8334
	Q9Y3A5	SBDS	51119
	P20340	RAB6A	5870
	Q6PGP7	TTC37	9652
	Q8WUJ3	KIAA1199	57214
	O00571	DDX3X	1654
	Q6NUK1	SLC25A24	29957
	Q9NZU5	LMCD1	29995
	P50281	MMP14	4323
	P60228	EIF3E	3646
	Q14192	FHL2	2274
	P49591	SARS	6301
	Q12931	TRAP1	10131
	Q86X55	CARM1	10498
	Q9UP95	SLC12A4	6560
	P21283	ATP6V1C1	528
	Q9UHQ9	CYB5R1	51706
	P05166	PCCB	5096
	P61353	RPL27	6155
	P51858	HDGF	3068

Proteins Uniquely identified in OA chondrocytes	Q16774	GUK1	2987
	Q5T5C0	STXBP5	134957
	Q92572	AP3S1	1176
	Q99470	SDF2	6388
	P61225	RAP2B	5912
	Q9Y316	MEMO1	51072
	Q7Z3B1	NEGR1	257194
	O43681	ASNA1	439
	P51553	IDH3G	3421
	Q9Y305	ACOT9	23597
	P36639	NUDT1	4521
	O14964	HGS	9146
	Q13283	G3BP1	10146
	Q9P2J5	LARS	51520
	P61020	RAB5B	5869
	Q08378	GOLGA3	2802
	O43143	DHX15	1665
	P45974	USP5	8078
	P62917	RPL8	6132
	P24844	MYL9	10398
	P55290	CDH13	1012
	Q9Y2Q3	GSTK1	373156
	P29692	EEF1D	1936
	Q9P2R7	SUCLA2	8803
	P30533	LRPAP1	4043
	P62191	PSMC1	5700
	Q96AE4	FUBP1	8880
	P30711	GSTT1	2952
	Q13488	TCIRG1	10312
	O15400	STX7	8417
	O14828	SCAMP3	10067
	Q6UW02	CYP20A1	57404
	P62829	RPL23	9349
	Q07955	SRSF1	6426
	O75431	MTX2	10651
	Q14444	CAPRIN1	4076
	Q9Y259	CHKB	1120
	Q9UNL2	SSR3	6747
	Q9ULC3	RAB23	51715
	P54619	PRKAG1	5571
	P31689	DNAJA1	3301
	Q6YN16	HSDL2	84263
	P27635	RPL10	6134
	Q9H993	C6orf211	79624
	P11172	UMPS	7372
	Q92692	PVRL2	5819

Proteins Uniquely identified in OA chondrocytes			
	Q9UHB6	LIMA1	51474
	O95168	NDUFB4	4710
	O43617	TRAPPC3	27095
	Q9BY32	ITPA	3704
	P61758	VBP1	7411
	Q9HBH5	RDH14	57665
	Q96EM0	L3HYPDH	112849
	Q9UHX1	PUF60	22827
	Q8NF37	LPCAT1	79888
	Q9H3H3	C11orf68	83638
	O96011	PEX11B	8799
	O00142	TK2	7084
	Q9NRN7	AASDHPPT	60496
	P50552	VASP	7408
	P09669	COX6C	1345
	P63208	SKP1	6500
	P36404	ARL2	402
	Q9BQ69	MACROD1	28992
	P55036	PSMD4	5710
	P51114	FXR1	8087
	Q96EU7	C1GALT1C1	29071
	Q9Y2S7	POLDIP2	26073
	P17302	GJA1	2697
	Q9HB90	RRAGC	64121
	Q96A33	CCDC47	57003
	Q8WXF1	PSPC1	55269
	O95297	MPZL1	9019
	Q6GMV2	SMYD5	10322
